# The lipid profile for the prediction of prednisolone treatment response in patients with inflammatory hand osteoarthritis: The HOPE study

**DOI:** 10.1016/j.ocarto.2021.100167

**Published:** 2021-04-22

**Authors:** Marieke Loef, Tariq O. Faquih, Johannes H. von Hegedus, Mohan Ghorasaini, Andreea Ioan-Facsinay, Féline P.B. Kroon, Martin Giera, Margreet Kloppenburg

**Affiliations:** a Department of Rheumatology, Leiden University Medical Center, the Netherlands; b Department of Clinical Epidemiology, Leiden University Medical Center, the Netherlands; c Center for Proteomics and Metabolomics, Leiden University Medical Center, the Netherlands

**Keywords:** Lipids, Metabolomics, Hand osteoarthritis, Prednisolone, Prediction, Treatment response

## Abstract

**Objective:**

To explore the use of lipidomics for prediction of prednisolone treatment response in patients with inflammatory hand osteoarthritis.

**Design:**

The Hand Osteoarthritis Prednisolone Efficacy (HOPE) study included patients (n ​= ​92) with symptomatic inflammatory hand osteoarthritis, fulfilling the ACR criteria. The present analyses comprised only patients randomized to prednisolone treatment (10 ​mg daily, n ​= ​40). Response to prednisolone treatment was defined according to the OARSI-OMERACT responder criteria at six weeks. Baseline blood samples were obtained non-fasted. Lipid species were quantified in erythrocytes with the Lipidyzer™ platform (Sciex). Oxylipins were analyzed in plasma using an in-house LC-MS/MS platform. Elastic net regularized regression was used to predict prednisolone treatment response based on common patient characteristics alone and including the patients’ lipid profile. ROC analyses with 1000 bootstrapped area under the curve (AUC) was used to determine the discriminatory accuracy of the models.

**Results:**

Among included patients, 78% fulfilled the OARSI-OMERACT responder criteria. From the general patient characteristics, elastic net selected baseline hand function as only predictor of treatment response, with an AUC of 0.78 (0.56; 0.97). Addition of lipidomics resulted in an AUC of 0.92 (0.78; 0.99) and 0.85 (0.65; 0.98) for inclusion of the Lipidyzer™ platform and oxylipin platform, respectively.

**Conclusion:**

Our results suggest that the patients’ lipid profile may improve the discriminative accuracy of the prediction of prednisolone treatment response in patients with inflammatory hand osteoarthritis compared to prediction by commonly measured patient characteristics alone. Hence, lipidomics may be a promising field for biomarker discovery for prediction of anti-inflammatory treatment response.

## 1 Introduction

Hand osteoarthritis (OA) is one of the most prevalent OA phenotypes, and it is associated with pain, stiffness, functional impairment and a loss in quality of life [[1], [2], [3], [4]]. Currently, there is a high unmet need for disease modifying drugs for the treatment of osteoarthritis (OA). The role of inflammation in hand OA and its association with pain [5,6] has sparked increasing interest for targeting inflammation in therapeutic research. To this regard, the Hand Osteoarthritis Prednisolone Efficacy (HOPE) study was set up. The HOPE study is a blinded, randomized placebo-controlled trial, that investigated the effect of prednisolone treatment in patients with painful, inflammatory hand OA. The HOPE study showed a clinically relevant decrease in pain in patients using prednisolone [7]. Since pharmacological treatments usually show marked variation in treatment response, it is important to carefully select patients who will most likely benefit from treatment, to maximize the desired therapeutic effect, and minimize overtreatment and potential adverse effects. Metabolomics may aid the identification of biomarkers of therapeutic responsiveness [8].

Lipids are essential for joint physiology [9,10]. However, to maintain normal physiology, a tight control of lipid species is warranted. In addition, various lipids and their metabolites are involved in pathophysiological settings, in particular in inflammation. Moreover, they have been shown to play an important role in inflammation in auto-immune diseases [11], as well as in OA [12,13]. Therefore, lipidomics, involving the identification and quantification of lipid metabolites, may be particularly relevant as biomarker of therapeutic responsiveness to anti-inflammatory medication. In addition, previous lipid profiling studies have suggested an altered lipid metabolism in patients with OA [[14], [15], [16]]. In particular, associations between differing levels of phospholipids and OA have been observed [[16], [17], [18]]. Hence, the patients’ lipid profile may be predictive of response to anti-inflammatory treatment in patients with inflammatory hand OA. To our knowledge, the use of lipidomics for prediction of treatment response in patients with OA has not previously been studied.

Therefore, we explored the patients’ lipid profile for the prediction of prednisolone treatment response in patients with inflammatory hand OA.

## 2 Methods

### 2.1 Study design

The HOPE study included patients with symptomatic hand OA, fulfilling the American College of Rheumatology criteria [19] and presenting signs of inflammation in the distal and proximal interphalangeal (DIP/PIP) joints. Full description of patient inclusion and procedures can be found elsewhere [7]. Briefly, patients were required to have: finger pain of ≥30 ​mm on a 100 ​mm visual analogue scale (VAS) and flaring upon 48-h NSAID washout (defined as ≥20 ​mm worsening), ≥4 DIP/PIP joints with osteoarthritic nodes, ≥1 DIP/PIP joints with soft swelling or erythema, and ≥1 DIP/PIP joints with positive power Doppler signal or synovitis grade ≥2 on ultrasound. Patients were excluded from participation in case of chronic inflammatory rheumatic diseases, psoriasis, uncontrolled serious comorbidities, malignancy, infectious disease, and immune modulating drug use within 90 days before baseline. Patients (n ​= ​92) were randomly assigned (1:1) to receive 10 ​mg prednisolone daily, or placebo, for six weeks. The present study comprised of patients randomized to prednisolone treatment only (n ​= ​40). Treatment adherence has been reported previously [7]. The HOPE study (Netherlands Trial Registry (NTR5263)) was approved by the local medical ethics committees and conducted in accordance with Good Clinical Practice guidelines and Declaration of Helsinki. All patients provided written informed consent.

### 2.2 Patient reported outcomes

At baseline and week six, patients completed a VAS for finger pain and VAS global assessment on a 0–100 ​mm scale, and the Australian/Canadian Hand Osteoarthritis Index (AUSCAN) pain (scored as 0–20) and function (scored as 0–36) subscales (higher scores are worse). At week six, fulfilment of the OMERACT-OARSI responder criteria was assessed, which was defined as a relative improvement ≥50% and absolute change ≥20/100 in AUSCAN pain or function, or a relative improvement ≥20% and absolute change ≥10/100 in ≥2 of the following: AUSCAN pain, AUSCAN function or VAS patient global assessment [20]. In the OMERACT-OARSI criteria, the AUSCAN pain and function subscale scores are used on a 0–100 scale. The AUSCAN pain and function subscale scores were rescaled from 0 to 20 and 0–36, respectively, to 0–100. We calculated absolute change as the baseline score minus the follow-up score, and relative change as the absolute change divided by the baseline score.

### 2.3 Baseline imaging

All interphalangeal and metacarpophalangeal joints were assessed on baseline radiographs of both hands (30 joints). Radiographic OA severity was investigated using the Kellgren and Lawrence (KL) grading system on a 0–4 scale [21]. Erosive OA was defined as having ≥1 joint in the erosive or remodelling phase according to the Verbruggen-Veys score [22]. Synovial thickening was assessed on ultrasound on a 0–3 scale [6]. A sum score adding the scores of all investigated joints was calculated for KL (0–120) and synovitis (0–90). The reliability of all scoring methods was good [7].

### 2.4 Lipidomics measurements

Blood samples were obtained non-fasted at baseline at various time points during the day in EDTA-tubes, following a standardized protocol. The blood samples were centrifuged for 10 ​min at 2200×*g* to separate plasma from the cellular fraction. Erythrocytes were isolated by ficoll density gradient centrifugation and washed 3x with PBS. Plasma samples were quenched using 600 ​μL MeOH (Honeywell, 349661 ​L), and 8 ​μL IS was added (containing: 500 ​pg/mL PGE2-d4, 5 ​ng/mL DHA-d5, 500 ​pg/mL LTB4-d4 and 500 ​pg/mL 15 ​S-HETE-d8). Samples were stored at −80 ​°C topped with argon until further analyses [23].

The Lipidyzer™ platform (Sciex) was used to quantify total lipid content in erythrocytes (nmol/mL). Lipid extraction was performed using methyl-tert-butylether as described by Matyash et al. with some modifications [24]. To 25 ​μL of erythrocyte sample the following was added: 160 ​μL MeOH, 50 ​μL internal standard solution (Lipidyzer™ internal standard kit, containing ​> ​50 labeled internal standards for 13 lipid classes), and 550 ​μL methyl-tert-butylether. Samples were vortexed and left at room temperature for 30 ​min. Subsequently, 200 ​μL water was added for phase separation and the samples were centrifuged at 13.100×*g*. The upper layer was transferred to a glass vial and lipid extraction was repeated by adding 300 ​μL methyl-tert-butylether, 100 ​μL MeOH and 100 ​μL water. The organic extracts were combined and dried under a gentle stream of nitrogen. Lipidyzer running buffer (250 ​μL) was added and samples were transferred to a glass vial with insert for injection. Briefly, the Lipidyzer platform is a flow-injection-based ion-mobility triple quadrupole system consisting of a Sciex 5500 QTrap equipped with SelexIon technology coupled to a Shimadzu Nexera series UHPLC system used for injection and delivering running buffer at 7 ​μL/min. Two methods were used for the injection of a total of 50 ​μL of the resuspended samples. First, PC, PE, (L)PC, (L)PE, and SM lipid classes were analyzed using method 1, operating with active DMS separation under the following conditions: DMS temperature low, modifier (propanol) composition low, separation voltage 3500 ​V, DMS resolution enhancement low. Next, FFA, TAG, DAG, CER, dihydroceramide (DCER), lactosylceramide (LCER), hexosylceramide (HCER), and CE lipids were analyzed applying method 2, for which the DMS cell was not activated. The MS operated under the following conditions: curtain gas 17, CAD gas medium, ion spray voltage 4100 ​V in ESI ​+ ​mode and −2500 ​V in ESI− mode, temperature 200 ​°C, nebulizing gas 17, and heater gas 25. Further technical detail can be found elsewhere [[25], [26], [27]]. Lipid concentrations were corrected for the erythrocyte protein pellet content, which was quantified using a Micro BCA Protein Assay Kit (Thermo Scientific, Waltham, MA, USA). Samples were measured in a randomized batch controlled fashion. The lipid concentrations were corrected for the erythrocyte protein pellet content. After preprocessing of the Lipidyzer™ data (Supplementary file, figure S1), 286 lipid species were available for further analyses (Supplementary file, table S1).

Oxylipins were measured in plasma, using liquid-chromatography combined with mass spectrometry (LC-MS/MS) analysis in negative electrospray ionization mode as described previously [28]. A QTrap 6500 mass spectrometer in negative ESI mode (Sciex, Nieuwerkerk aan den Ijssel, The Netherlands) was used, coupled to a LC system employing LC-30AD pumps, a SIL-30AC auto sampler, and a CTO-20AC column oven (Shimadzu, ‘s-Hertogenbosch, The Netherlands). A Kinetex C18 50 ​× ​2.1 ​mm, 1.7 ​μm column, combined with a C8 pre column (Phenomenex, Utrecht, The Netherlands) was used, kept at 50 ​°C. A gradient of water and Methanol with 0.01% acetic acid was used. An injection volume of 40 ​μL was used, with a flow rate of 400 ​μL/min [34]. Oxylipins were identified using characteristic mass transitions and relative retention times. Only peaks with a signal to noise >10 were included, resulting in identification of 25 oxylipins. For a subset of these, synthetic standards were available, allowing for quantification (ng/mL). Area ratios were calculated for all other oxylipins.

### 2.5 Statistical analyses

Descriptive statistics were used for baseline patient characteristics. Two-sample t-tests and Chi-square tests were used as appropriate to assess differences in baseline general patient characteristics. We used elastic net (EN) regularized regression for selection of predictors [29]. EN uses an additional tuning parameter (alpha) to combine the properties of ridge regression and lasso by applying both L1 and L2 penalties. Thereby, it simultaneously performs automatic variable selection and continuous shrinkage, while also dealing with high correlations amongst predictors. Prior to fitting the model, lipid measurements below the detection limit were imputed with the minimum measured value divided by two, all lipid variables were logarithmically transformed due to a non-normal distribution, and were mean scaled to ensure comparability by giving the metabolites equal weight. We performed EN regularization with a logit model, defining the OARSI-OMERACT responder status as the outcome. Prior to fitting the EN models, we performed a 10-fold cross-validation (CV) for selection of the optimal tuning parameters based on the smallest CV mean prediction error. In addition, we used manual alpha selection based on the out-of-sample deviance ratio and CV mean deviance to investigate the performance of more comprehensive models. First, a model was fit with commonly assessed patient characteristics and patient reported outcomes, measured at baseline (model 1). Second, we fitted model 2 by adding the Lipidyzer™ platform lipids to model 1. Third, we fitted model 3 by adding the oxylipins to model 1. Fourth, we combined the general patient characteristics with both lipid platforms in model 4. Lastly, we fitted a model with the predictors selected by model 2 and 3. We used the Stata command: *elasticnet logit depvar othervars, alpha(0.1(0.1)1) selection(cv, fold(10) alllambdas)*. The discriminatory accuracy of the model was estimated by receiver operating characteristic (ROC) analyses (Stata command: *rocreg*). The area under the curve (AUC) and corresponding 95% confidence intervals (CI) were calculated using 1000 bootstrap replications. Additionally, we performed sensitivity analyses investigating the association between the lipid predictors and treatment response using univariable logistic regression. Stata V16.1 (StataCorp LP, TX, USA) was used for all analyses.

## 3 Availability of data and materials

The data underlying this article cannot be shared publicly due to the privacy of the participants of the HOPE study and legal reasons (HOPE study participants did not sign informed consent to make their data publicly available). The data is available upon request to interested qualified researchers. Data requests should be sent to the corresponding author.

## 4 Results

### 4.1 Study population

**Fig. 1:**
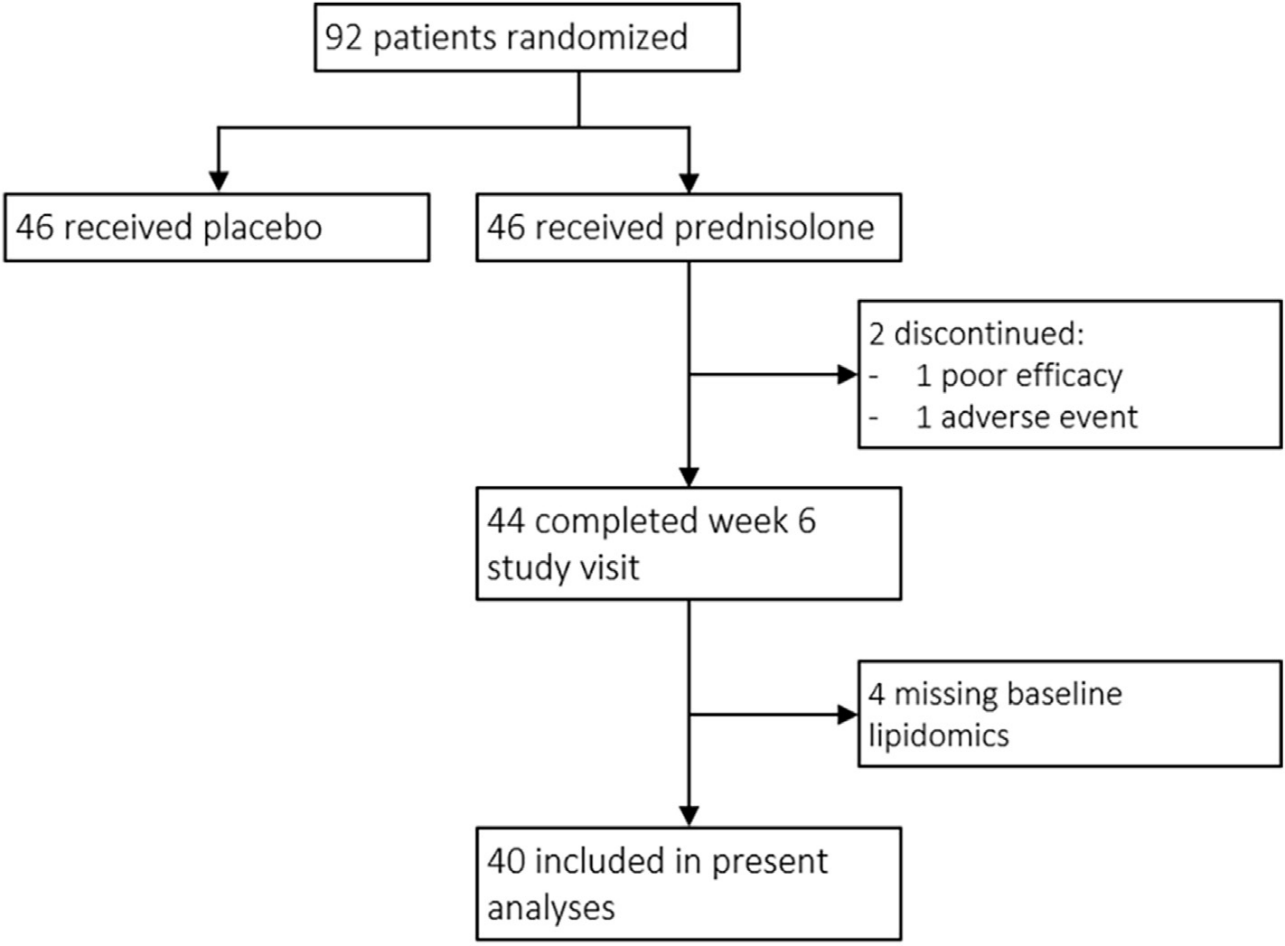
Flowchart of patient numbers The present analyses included only patients randomized to prednisolone treatment. Of the 46 patients assigned, 2 discontinued the study due to poor efficacy or an adverse event. Four patients were excluded due to missing lipid measurements at baseline.

Baseline lipid measurements and the OARSI-OMERACT responder status at week six were available in 40 prednisolone-treated patients. Fig. 1 shows a flowchart of included patients. Of these patients, 31 (78%) fulfilled the OARSI-OMERACT responder criteria. The percentage of patient fulfilling either the major criteria or a particular combination of minor criteria is presented in supplementary figure 2. Patients responding to prednisolone treatment showed statistically worse baseline AUSCAN function scores (19.6 ​± ​6.6) than non-responders (11 ​± ​7.5). None of the other general characteristics differed between responders and non-responders (Table 1).

**Table 1:** Baseline characteristics of prednisolone-treated patients in the HOPE study.

	All prednisolone treated n ​= ​40	Responders n ​= ​31 (78%)	Non-responders n ​= ​9 (23%)
**General characteristics**			
Age, year	62.4 (9.3)	62.9 (9.4)	60.8 (9.4)
Sex, % women	85	84	89
BMI, kg/m^2^	27.4 (4.4)	27.8 (4.2)	26.2 (5.0)
Education, % high	46	42	56
Disease duration	6.7 (7.1)	7.2 (7.4)	4.9 (5.8)
Erosive OA, %	71	74	56
Kellgren-Lawrence sum score, 0-120	35.1 (16.4)	34.1 (16.5)	37.5 (14.7)
Ultrasound synovitis sum score, 0-90	16.2 (6.6)	15.5 (6.4)	18.7 (7.2)
VAS global assessment, 0-100	52.3 (20.6)	54.2 (16.8)	45.6 (30.8)
AUSCAN pain, 0-20	11.0 (3.3)	11.3 (2.4)	10 (5.4)
AUSCAN function, 0-36	17.7 (7.6)	19.6 (6.6)	11 (7.5)

Numbers represent mean (SD) unless otherwise specified. Abbreviations: AUSCAN ​= ​Australian/Canadian Hand Osteoarthritis Index, BMI ​= ​body mass index, VAS ​= ​visual analogue scale.

### 4.2 Prediction of treatment response using general patient characteristics

**Fig. 2:**
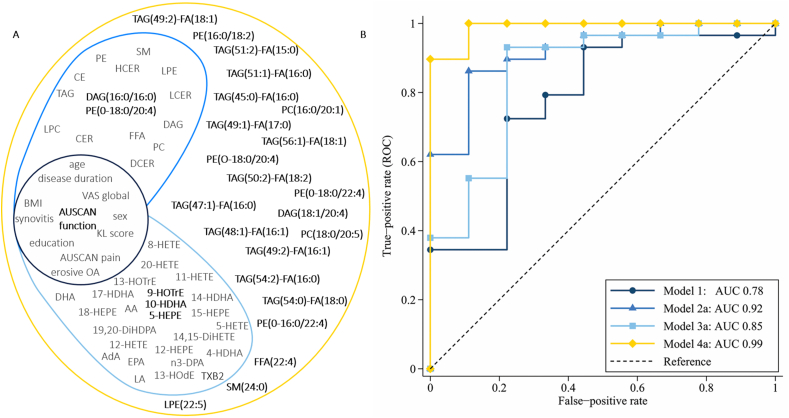
Prediction model characteristics. A) shows the variables included for model fitting of the three prediction models, colours correspond to the lines of the ROC curves in B). Of model 2, only the lipid classes are shown. Variables in bold font were selected in the final models. Model 1: General patient characteristics, model 2: model 1 ​+ ​Lipidyzer™ platform, model 3: model 1 ​+ ​oxylipin platform, model 4: all variables included. Abbreviations: AUC ​= ​area under the curve, AUSCAN ​= ​Australian/Canadian Hand Osteoarthritis Index, CE ​= ​cholesteryl ester, CER ​= ​ceramide, DAG ​= ​diacylglycerol, DCER ​= ​dihydroceramide, FFA ​= ​free fatty acid, HCER ​= ​hexosylceramide, KL = Kellgren-Lawrence, LCER ​= ​lactosylceramide, (L)PC = (lyso)phosphatidylcholines, (L)PE = (lyso)phosphatidylethanolamine, OA ​= ​osteoarthritis, SM ​= ​sphingomyelin, TAG ​= ​triacylglycerol, VAS ​= ​visual analogue scale, 9-HOTrE ​= ​9-hydroxy-octadecatrienoic acid, 5-HEPE ​= ​5-hydroxy-eicosapentaenoic acid, 10-HDHA ​= ​10-hydroxy-docosahexaenoic acid. (For interpretation of the references to colour in this figure legend, the reader is referred to the Web version of this article.)

The general characteristics presented in Table 1 were entered in model 1 as predictors of OARSI-OMERACT responder status. Only AUSCAN function was selected in the model (worse function associated with response), resulting in an AUC with 95% CI of 0.78 (0.56; 0.94). Predictors entered in the model, predictors selected by EN, and corresponding ROC curves of the models are shown in Fig. 2. Table 2 presents the baseline concentrations of the selected lipids. Tuning parameters and model deviances of all models are provided in Table 3.

**Table 2:** Baseline levels of selected lipids.

	All prednisolone treated n ​= ​40	Responders n ​= ​31 (78%)	Non-responders n ​= ​9 (23%)
**Levels selected Lipidyzer**^**TM**^**lipids**			
DAG(16:0/16:0), nmol/mL	0.28 (0.12)	0.30 (0.12)	0.18 (0.084)
PE(O-18:0/20:4), nmol/mL	66.01 (12.20)	63.26 (10.65)	75.48 (13.04)
**Levels selected oxylipins**			
9-HOTrE, area ratio	0.12 (0.09)	0.093 (0.059)	0.20 (0.14)
5-HEPE, area ratio	0.011 (0.015)	0.014 (0.016)	0.0043 (0.0032)
10-HDHA, ng/mL	0.0039 (0.0044)	0.0046 (0.0048)	0.0019 (0.0020)

Numbers represent mean (SD). Abbreviations: DAG ​= ​diacylglycerol, PE ​= ​phosphatidylethanolamine, 9-HOTrE ​= ​9-hydroxy-octadecatrienoic acid, 5-HEPE ​= ​5-hydroxy-eicosapentaenoic acid, 10-HDHA ​= ​10-hydroxy-docosahexaenoic acid.

**Table 3:** Selected predictors and prediction model parameters.

	Selected predictors		Tuning parameters *Alpha Lambda*		Out-of-sample deviance ratio	CV mean deviance	AUC (95% CI)
**Model 1** General characteristics	1	AUSCAN function	1.00	0.113	0.0838	0.9770132	0.78 (0.56; 0.94)
**Model 2** Model 1 ​+ ​Lipidyzer™	301	All variables^a^	0	23.327	−0.0269	1.095027	0.95 (0.85; 0.99)
**Model 2a** Model 1 ​+ ​Lipidyzer™ *Manual alpha selection*	3	AUSCAN function DAG(16:0/16:0) PE(O-18:0/20:4)	1.00	0.150	−0.0275	1.095633	0.92 (0.78; 0.99)
**Model 3** Model 1 ​+ ​oxylipins	40	All variables^a^	0	2.193	−0.0689	1.170265	0.88 (0.73; 0.97)
**Model 3a** Model 1 ​+ ​oxylipins *Manual alpha selection*	4	AUSCAN function 9-HOTrE 5-HEPE 10-HDHA	0.60	0.182	−0.0835	1.186297	0.85 (0.65; 0.98)
**Model 4** All variables combined	326	All variables^a^	0	8.853	−0.0505	1.150134	0.97 (0.90; 1)
**Model 4a** All variables combined *Manual alpha selection*	27	AUSCAN function DAG(16:0/16:0) DAG(18:1/20:4) FFA(22:4) LPE(22:5) PC(16:0/20:1) PC(18:0/20:5) PE(16:0/18:2) PE(O-16:0/22:4) PE(O-18:0/20:4) PE(O-18:0/22:4) SM(24:0) TAG(45:0)-FA(16:0) TAG(47:1)-FA(16:0) TAG(48:1)-FA(16:1) TAG(49:1)-FA(17:0) TAG(49:2)-FA(16:1) TAG(49:2)-FA(18:1) TAG(50:2)-FA(18:2) TAG(51:1)-FA(16:0) TAG(51:2)-FA(15:0) TAG(54:0)-FA(18:0) TAG(54:2)-FA(16:0) TAG(56:1)-FA(18:1) 9-HOTrE 5-HEPE 10-HDHA	0.2	0.475	−0.0921	1.195637	0.99 (0.93; 1)
**Model 5** *Predefined model based on predictor selection of model 2a and 3a*	6	AUSCAN function DAG(16:0/16:0) PE(O-18:0/20:4) 9-HOTrE 5-HEPE 10-HDHA	0	0.079	0.2993	.7671022	0.95 (0.81; 1)

a See additional file 1, tables A1 and A2 for the included lipids. Abbreviations: AUSCAN ​= ​Australian/Canadian Hand Osteoarthritis Index, AUC ​= ​area under the curve, CI ​= ​confidence interval, CV ​= ​cross-validation, DAG ​= ​diacylglycerol, FFA ​= ​free fatty acid, (L)PE = (lyso)phosphatidylethanolamine, PC ​= ​phosphatidylcholine, SM ​= ​sphingomyelin, TAG ​= ​triacylglycerol, 9-HOTrE ​= ​9-hydroxy-octadecatrienoic acid, 5-HEPE ​= ​5-hydroxy-eicosapentaenoic acid, 10-HDHA ​= ​10-hydroxy-docosahexaenoic acid.

### 4.3 Added value of lipidomics for prediction of treatment response - Lipidyzer™

In model 2, we added the 286 Lipidyzer™ platform lipid species to model 1. Cross-validated parameter tuning selected an alpha of 0, resulting in the inclusion of all predictors in the model with an AUC of 0.95 (0.85; 0.99). With only minor increase in deviance (CV mean deviance 1.096 vs 1.095), a model (2a) with an alpha of 1 resulted in the selection of three variables: AUSCAN function and two lipids: diacylglycerol(DAG)(16:0/16:0) (higher levels associated with response), and phosphatidylethanolamine(PE)(O-18:0/20:4) (lower levels associated with response), with an AUC of 0.92 (0.78; 0.99).

### 4.4 Added value of lipidomics for prediction of treatment response – oxylipins

In model 3, the 25 identified oxylipins were added to model 1. With automated parameter tuning an alpha of 0 was used, selecting all variables for the model, resulting in an AUC of 0.88 (0.73; 0.97). However, with only marginal inflation of the CV mean deviance (1.186 vs 1.184) a more comprehensible model (3a) could be fit, which included AUSCAN function and three oxylipin predictors: 9-hydroxy-octadecatrienoic acid (HOTrE) (lower levels associated with response), 5-hydroxy-eicosapentaenoic acid (HEPE) and 10-hydroxy-docosahexaenoic acid (HDHA) (higher levels associated with response), with an AUC of 0.85 (0.65; 0.98).

### 4.5 Combining all predictors

Lastly, we combined the general patient characteristics with both lipid platforms in model 4. Again, automated parameter tuning resulting in an alpha of 0. Including all 326 variables in the model resulted in an AUC of 0.97 (0.90; 1). A more comprehensive model (4a) could be fit using an alpha of 0.2, resulting in the selection of 27 predictors. This model included all previously selected predictors from models 2 and 3, as well as 21 additional higher order (Lipidyzer™) lipids (Table 3), resulting in a model with an AUC of 0.99 (0.93; 1). In addition, we ran model 5 in which we included only the 6 predictors previously selected by EN in models 2 and 3. The discriminative ability of this model was only slightly less compared to the full model, with an AUC of 0.95 (0.81; 1), and significantly improved the prediction compared to a model based on general patient characteristics alone (model 1 vs model 5, p ​= ​0.03).

### 4.6 Sensitivity analyses

The univariable associations of baseline lipid levels with prednisolone treatment response are shown in the supplementary file, tables S1 and S2. The lipids included in model 2 and 3 were univariably among the lipids most strongly associated with treatment response, supporting the selection of predictors by the EN models.

## 5 Discussion

In this exploratory study we investigated the patients’ lipid profile for the prediction of prednisolone treatment response in patients with painful inflammatory hand OA. We showed that lipidomics improved the discriminative accuracy of the prediction, when compared to commonly measured patient outcomes alone. Our results suggest that lipidomics is a promising field for further biomarker discovery for the prediction of anti-inflammatory treatment response.

The added predictive value of lipidomics is an interesting finding. From the Lipidyzer™ platform, lipids containing fatty acid chains of palmitic acid (16:0), stearic acid (18:0) and arachidonic acid (20:4) were selected as predictors. Palmitic acid is the most abundant saturated fatty acid (SFA) in humans; under physiological conditions its concentration is tightly controlled by desaturation to palmitoleic acid and oleic acid, or elongation to stearic acid [30]. Pathophysiological conditions may increase SFA content, leading to activation of toll-like receptor (TLR)-4 triggered inflammatory signalling cascades via nuclear factor kappa B (NFκB) and cyclooxygenase (COX)-2, increasing proinflammatory cytokine production [31]. Arachidonic acid, an omega-6 polyunsaturated fatty acid (PUFA), is the main precursor of proinflammatory eicosanoids, although it may also give rise to anti-inflammatory mediators. In addition, hydroxylation of the omega-3 PUFAs eicosapentaenoic acid (EPA) and docosahexaenoic acid (DHA) may lead to hydroxyeicosapentaenoic acids (HEPE) and hydroxydocosahexaenoic acids (HDHA), which are precursors of anti-inflammatory and pro-resolving mediators [32]. Possibly, the lipid profile represents an indication of the patients’ inflammatory state, and their likelihood to respond to anti-inflammatory treatment. However, we should be careful to avoid causal interpretations of our results since no causal inferences can be drawn from prediction analyses.

Furthermore, our results suggest that amongst other patient characteristics such as pain, radiographic OA severity and synovitis, hand function is the most contributing to the prediction of treatment response. Despite possible influences of the small sample size and patient selection, which likely resulted in a lack of predictive ability of characteristics such as age and sex, as well as regression to the mean, it implies that patients’ hand function may be an important outcome to consider when making treatment decisions.

To our knowledge lipidomics for the prediction of treatment response in hand OA has not previously been investigated. A major strength of our study is the use of high-quality trial data. Furthermore, we have used lipidomics data from two different platforms, the standardized and commercially available Lipidyzer™ platform for the measurement of a large variety of higher order lipids, and an in-house developed platform for the measurement of oxylipins.

However, there are also limitations to our study. Most notable is the small sample, which has likely resulted in overfitting of the models and a higher degree of uncertainty of the estimations. Also, since no study population with comparable data was available, external validation was not possible. In addition, the analyses have been performed in a specific, carefully selected patient population, therefore results may not be generalizable to other patient populations. The blood samples were obtained non-fasted at variable time points during the day due to differences in scheduled hospital visits. Although this may be viewed as a limitation, this procedure is a good reflection of daily practice and limits patient burden. Moreover, the identification of predictions for treatment response that do not required fasted or strictly scheduled sampling will benefit the feasibility and implementation in clinical practice. However, this may have resulted in additional variability in the lipid measurements. In a recent study by our research group we described intra-day variability (ICC) of (DAG)(16:0/16:0) of 0.62 and of (PE)(O-18:0/20:4) ICC of 0.46 [33], representing moderate to good reproducibility of the lipids selected in model 2a. Furthermore, we cannot exclude *in vitro* auto-oxidation of lipid metabolites. However, as this would have occurred to a similar extend in responders and non-responders, it is unlikely this has influenced our findings. Hence, the use of lipidomics, and in particular the development of a lipid biomarker, for the prediction of prednisolone treatment response warrants further investigation.

In conclusion, this exploratory study suggests that lipidomics may prove valuable in the prediction of prednisolone treatment response in patients with inflammatory hand OA. Prediction of treatment response may aid the selection of patients with a high likelihood of treatment benefit, which is crucial to prevent overtreatment and unnecessary exposure to adverse effects.

## Contributions

MK was the principle investigator. ML analyzed the data and drafted the article. All authors contributed to the design of the study, data interpretation and critically revising of the article. All authors give final approval of the submitted article.

## Role of the funding source

The HOPE study was funded by the Dutch Arthritis Foundation under grant HOPE 14-1-303. The funding source had no role in design of the study, data collection, analyses or interpretation, or in writing of the manuscript.

## Declaration of competing interest

ML and MK report support from the 10.13039/501100010767Innovative Medicines Initiative Joint Undertaking under Grant Agreement n° 115,770, paid to the institution. In addition, MK reports research grants from the Dutch Arthritis Society, Pfizer, consultancy fees from AbbVie, Pfizer, Levicept, GlaxoSmithKline, and Merck Serono, and royalty fees from UpToDate, all paid to her department, and she was the local investigator of an industry-driven trial (run by AbbVie). MGi received support from the H2020 ITN grant ArthritisHeal (#812890) and the 10.13039/501100003246NWO project 184.034.019. MG is an early stage researcher in the 10.13039/100010662H2020 funded EU ITN project 371 ArthritisHeal (#812890). All other authors declare no competing interests.
