# Supplementary material for: The lipid profile for the prediction of prednisolone treatment response in patients with inflammatory hand osteoarthritis: The HOPE study
Source: Osteoarthr Cartil Open. 2021 Apr 22;3(4):100167. doi: 10.1016/j.ocarto.2021.100167 (PMC9718086; doi:10.1016/j.ocarto.2021.100167)
Supplement: Multimedia component 1 [file mmc1.docx]

**
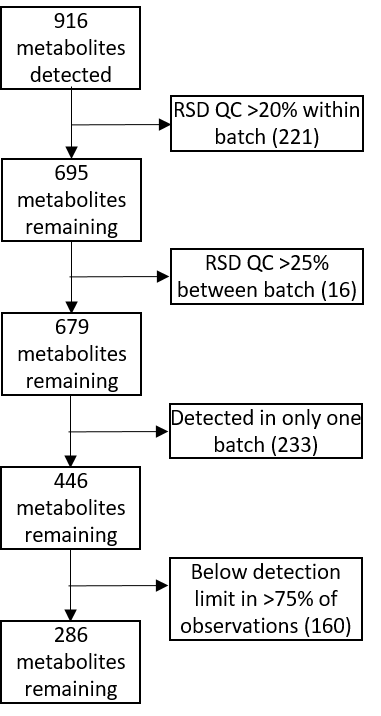
**

**Figure S1. Pre-processing steps of Lipidyzer^TM^ variables**
Lipid variables were excluded if the relative standard deviation (RSD) of the quality control (QC) was >20% within a batch, or >25% between separate batches, if a lipid was detected in one batch only, or if below the detection limit in >75% of the observations.

**
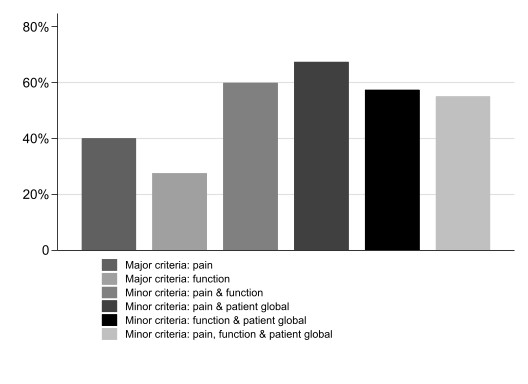
**

**Figure S2. Percentage of patient fulfilling responder criteria sets**.

Responders were defined according to the OARSI-OMERACT responder criteria, which was defined as a relative improvement ≥50% and absolute change ≥20/100 in AUSCAN pain or function (major criteria), or a relative improvement ≥20% and absolute change ≥10/100 in ≥2 of the following (minor criteria): AUSCAN pain, AUSCAN function or VAS patient global assessment.

**Table S1**. Association between Lipidyzer^TM^ lipid levels and prednisolone treatment response

| **Lipid** | **OR (95% CI)** | **P-value** |
| --- | --- | --- |
| DAG(16:0/16:0) | 3.09 (1.27; 7.52) | 0.013 |
| TAG(49:2)-FA(16:1) | 3.35 (1.24; 9.07) | 0.017 |
| PE(O-18:0/20:4) | 0.25 (0.08; 0.81) | 0.020 |
| TAG(45:0)-FA(16:0) | 2.77 (1.15; 6.70) | 0.023 |
| TAG(48:1)-FA(16:1) | 2.51 (1.11; 5.69) | 0.027 |
| DAG(18:1/20:4) | 0.11 (0.01; 0.82) | 0.032 |
| PE(O-18:0/22:4) | 0.35 (0.13; 0.93) | 0.034 |
| TAG(54:0)-FA(18:0) | 2.12 (1.03; 4.39) | 0.042 |
| TAG(50:2)-FA(18:2) | 2.27 (91.03; 5.00) | 0.042 |
| TAG(51:1)-FA(16:0) | 2.77 (1.03; 7.41) | 0.043 |
| PE(O-16:0/22:4) | 0.35 (0.13; 0.98) | 0.045 |
| TAG(56:1)-FA(18:1) | 1.97 (1.00; 3.85) | 0.049 |
| TAG(49:1)-FA(18:1) | 3.46 (0.95; 12.64) | 0.060 |
| TAG(47:1)-FA(16:0) | 3.31 (0.93; 11.78) | 0.065 |
| TAG(50:2)-FA(16:1) | 2.25 (0.93; 5.43) | 0.070 |
| TAG(51:1)-FA(18:1) | 2.57 (0.92; 7.17) | 0.072 |
| LPE(22:5) | 2.65 (0.90; 7.80) | 0.077 |
| PE(P-16:0/20:3) | 1.87 (0.93; 3.76) | 0.079 |
| TAG(47:1)-FA(15:0) | 3.16 (0.86; 11.54) | 0.082 |
| TAG(52:2)-FA(18:0) | 2.20 (0.90; 5.40) | 0.084 |
| FFA(22:4) | 2.68 (0.88; 8.23) | 0.084 |
| PC(18:0/20:5) | 2.34 (0.89; 6.16) | 0.086 |
| DAG(18:0/18:2) | 0.44 (0.17; 1.12) | 0.086 |
| SM(24:0) | 0.47 (0.20; 1.12) | 0.087 |
| FFA(16:1) | 3.99 (90.81; 19.64) | 0.088 |
| PE(18:0/20:4) | 0.52 (0.24; 1.12) | 0.095 |
| TAG(46:1)-FA(16:1) | 2.12 (0.86; 5.21) | 0.101 |
| TAG(48:2)-FA(18:1) | 2.72 (0.82; 9.00) | 0.102 |
| TAG(49:1)-FA(16:0) | 2.08 (0.85; 5.08) | 0.106 |
| TAG(45:0)-FA(15:0) | 2.07 (0.86; 5.01) | 0.106 |
| TAG(52:3)-FA(16:1) | 2.04 (0.85; 4.86) | 0.109 |
| PE(16:0/18:2) | 0.47 (0.19; 1.19) | 0.110 |
| TAG(544)-FA(18:1) | 2.06 (90.84; 5.02) | 0.113 |
| TAG(491)-FA(16:1) | 2.18 (0.83; 5.70) | 0.114 |
| PE(P-18:1/20:3) | 1.74 (0.88; 3.46) | 0.114 |
| FFA(14:0) | 3.23 (0.75; 13.89) | 0.116 |
| TAG(52:0)-FA(16:0) | 1.74 (0.87; 3.46) | 0.117 |
| TAG(54:5)-FA(18:1) | 2.08 (0.83; 5.21) | 0.118 |
| PE(P-18:0/20:3) | 1.74 (0.87; 3.50) | 0.118 |
| PC(16:0/22:5) | 1.74 (0.87; 3.49) | 0.119 |
| TAG(48:2)-FA(14:0) | 2.79 (0.76; 10.28) | 0.123 |
| TAG(50L1)-FA(16:1) | 4.23 (0.67; 26.62) | 0.124 |
| LPE(20:3) | 2.61 (0.77; 8.93) | 0.125 |
| PE(18:2/20:4) | 0.56 (0.26; 1.18) | 0.126 |
| SM(26:1) | 1.89 (0.83; 4.29) | 0.128 |
| PE(P-18:0/16:0) | 0.51 (0.21; 1.22) | 0.132 |
| TAG(56:4)-FA(20:4) | 2.51 (0.76; 8.30) | 0.132 |
| TAG(50:0)-FA(16:0) | 1.87 (0.83; 4.22) | 0.133 |
| FFA(20:2) | 3.20 (0.70; 14.65) | 0.134 |
| TAG(54:1)-FA(18:0) | 1.83 (0.82; 4.09) | 0.138 |
| LPC(20:4) | 2.10 (0.79; 5.57) | 0.138 |
| TAG(50:3)-FA(16:1) | 1.84 (0.82; 4.13) | 0.139 |
| PE(P-18:1/20:5) | 1.65 (0.85; 3.21) | 0.140 |
| TAG(54:3)-FA(16:0) | 1.98 (0.80; 4.94) | 0.141 |
| SM(24:1) | 1.89 (0.80; 4.44) | 0.145 |
| PC(14:0/20:3) | 0.46 (0.16; 1.31) | 0.148 |
| TAG(44:1)-FA(18:1) | 0.46 (0.16; 1.32) | 0.148 |
| PC(18:2/18:2) | 0.50 (0.20; 1.28) | 0.149 |
| CE(16:0) | 1.80 (0.81; 3.98) | 0.149 |
| FFA(24:0) | 2.19 (0.75; 6.38) | 0.150 |
| FFA(22:2) | 3.91 (0.59; 26.10) | 0.159 |
| PC(16:0/20:1) | 1.53 (0.84; 2.79) | 0.162 |
| TAG(48:0)-FA(16:0) | 2.06 (0.75; 5.70) | 0.163 |
| TAG(56:4)-FA(18:1) | 1.87 (0.78; 4.49) | 0.163 |
| TAG(56:7FA22:6) | 1.84 (0.78; 4.33) | 0.165 |
| TAG(46:2)-FA(16:1) | 1.90 (0.76; 4.74) | 0.167 |
| TAG(52:2)-FA(16:1) | 3.22 (0.61; 17.06) | 0.170 |
| TAG(54:4)-FA(18:0) | 1.66 (0.80; 3.43) | 0.173 |
| PC(18:0/22:6) | 1.85 (0.76; 4.52) | 0.174 |
| PE(18:1/18:2) | 0.62 (0.31; 1.24) | 0.175 |
| PE(P-18:1/18:2) | 0.54 (0.22; 1.32) | 0.178 |
| PE(P-18:0/20:4) | 0.60 (0.28; 1.27) | 0.178 |
| TAG(51:3)-FA(18:2) | 2.39 (0.67; 8.51) | 0.179 |
| TAG(50:1)-FA(18:1) | 1.88 (0.74; 4.78) | 0.182 |
| TAG(54:5)-FA(20:4) | 1.66 (0.78; 3.50) | 0.185 |
| TAG(48:3)-FA(16:1) | 1.76 (0.76; 4.05) | 0.187 |
| TAG(47:0)-FA(15:0) | 1.66 (0.78; 3.51) | 0.188 |
| TAG(48:2)-FA(16:1) | 1.92 (0.72; 5.15) | 0.193 |
| CE(16:1) | 1.84 (0.73; 4.63) | 0.196 |
| TAG(47:0)-FA(16:0) | 1.61 (0.78; 3.31) | 0.197 |
| TAG(48:2)-FA(16:0) | 1.81 (0.73; 4.47) | 0.197 |
| PE(P-18:1/20:4) | 0.58 (0.25; 1.34) | 0.204 |
| TAG(47:1)-FA(14:0) | 2.14 (0.66; 6.89) | 0.204 |
| PE(O-16:0/20:3) | 1.53 (0.79; 2.96) | 0.206 |
| TAG(54:2)-FA(20:1) | 2.05 (0.67; 6.28) | 0.210 |
| PC(18:2/20:4) | 0.59 (0.26; 1.35) | 0.215 |
| HCER(24:1) | 1.53 (0.78; 2.99) | 0.218 |
| TAG(49:2)-FA(16:0) | 2.13 (0.64; 7.12) | 0.219 |
| PC(18:2/16:1) | 0.62 (0.29; 1.33) | 0.219 |
| TAG(46:0)-FA(14:0) | 1.58 (0.76; 3.30) | 0.223 |
| FFA(20:5) | 1.49 (0.78; 2.85) | 0.231 |
| PC(18:1/18:3) | 0.63 (0.29; 1.35) | 0.234 |
| TAG(46:0)-FA(16:0) | 1.75 (0.69; 4.46) | 0.242 |
| PC(16:0/20:5) | 1.69 (0.70; 4.09) | 0.243 |
| TAG(49:3)-FA(16:1) | 2.03 (0.62; 6.67) | 0.245 |
| PE(18:1/22:5) | 1.55 (0.74; 3.27) | 0.247 |
| PE(O-18:0/20:3) | 1.51 (0.74; 3.08) | 0.252 |
| PC(18:1/18:2) | 0.61 (0.27; 1.41) | 0.252 |
| TAG(49:0)-FA(17:0) | 1.74 (0.67; 4.53) | 0.253 |
| PE(P-18:1/18:1 | 0.61 (0.26; 1.43) | 0.256 |
| TAG(44:1)-FA(16:1) | 1.88 (0.63; 5.62) | 0.257 |
| TAG(55:4)-FA(18:1) | 1.65 (0.68; 4.00) | 0.265 |
| TAG(51:1)-FA(17:0) | 1.61 (0.69; 3.73) | 0.267 |
| TAG(52:4)-FA(16:1) | 1.70 (0.66; 4.35) | 0.272 |
| PE(P-16:0/22:4) | 0.65 (0.30; 1.40) | 0.272 |
| TAG(48:1)-FA(16:0) | 1.62 (0.68; 3.87) | 0.278 |
| TAG(50:2)-FA(18:1) | 1.57 (0.69; 3.56) | 0.281 |
| PE(18:1/22:6) | 1.55 (0.70; 3.46) | 0.281 |
| PC(18:1/20:5) | 1.49 (0.72; 3.11) | 0.282 |
| PC(18:1/20:3) | 0.64 (0.28; 1.45) | 0.284 |
| FFA(22:5) | 1.52 (0.71; 3.28) | 0.285 |
| TAG(50:2)-FA(14:0) | 1.77 (0.62; 5.08) | 0.287 |
| DAG(18:1/18:2) | 0.56 (0.19; 1.63) | 0.288 |
| TAG(54:4)-FA(18:2) | 1.56 (0.69; 3.51) | 0.288 |
| HCER(24:0) | 0.65 (0.29; 1.45) | 0.289 |
| TAG(56:8-FA(20:4) | 0.66 (0.30; 1.43) | 0.290 |
| LCER(16:0) | 1.53 (0.69; 3.39) | 0.292 |
| PE(16:0/18:1) | 0.66 (0.30; 1.43) | 0.295 |
| CER(24:1) | 1.48 (0.71; 3.11) | 0.296 |
| PC(18:0/18:3) | 1.47 (0.71; 3.02) | 0.298 |
| TAG(50:3)-FA(18:1) | 1.48 (0.70; 3.10) | 0.303 |
| TAG(49:2)-FA(15:0) | 1.59 (0.66; 3.85) | 0.304 |
| LPC(18:2) | 0.60 (0.22; 1.60) | 0.305 |
| PE(O-16:0/18:2) | 0.64 (0.27; 1.53) | 0.311 |
| TAG(52:4)-FA(18:1) | 1.48 (0.69; 3.19) | 0.313 |
| TAG(54:2)-FA(18:2) | 1.53 (0.66; 3.54) | 0.319 |
| TAG(54:3)-FA(18:2) | 1.38 (0.73; 2.59) | 0.324 |
| TAG(52:1)-FA(18:1) | 1.52 (0.66; 3.51) | 0.324 |
| PE(P-18:0/22:4) | 0.68 (0.31; 1.47) | 0.324 |
| TAG(48:1)-FA(18:1) | 1.45 (0.67; 3.12) | 0.341 |
| DAG(16:0/18:1) | 1.53 (0.64; 3.66) | 0.342 |
| PE(P-18:0/18:2) | 0.68 (0.30; 1.52) | 0.342 |
| TAG(42:0)-FA(16:0) | 1.51 (0.65; 3.51) | 0.343 |
| PE(O-16:0/20:4) | 0.68 (0.30; 1.52) | 0.345 |
| TAG(46:1)-FA(18:1) | 0.71 (0.34; 1.47) | 0.354 |
| TAG(50:1)-FA(16:0) | 1.49 (0.64; 3.44) | 0.356 |
| TAG(52:2)-FA(18:1) | 1.48 (0.64; 3.42) | 0.360 |
| TAG(56:6)-FA20:4) | 1.48 (0.64; 3.44) | 0.360 |
| TAG(54:6)-FA18:2) | 1.45 (0.65; 3.25) | 0.365 |
| TAG(46:1)-FA(14:0) | 1.46 (0.64; 3.29) | 0.366 |
| CE(18:2) | 0.74 (0.38; 1.43) | 0.368 |
| FFA(18:1) | 1.43 (0.65; 3.12) | 0.370 |
| TAG(54:3)-FA(18:0) | 1.41 (0.64; 3.10) | 0.393 |
| TAG(56:4)-FA(18:0) | 1.37 (0.66; 2.84) | 0.394 |
| TAG(52:3)-FA(18:2) | 1.42 (0.63; 3.22) | 0.399 |
| TAG(50:3)-FA(18:2) | 1.42 (0.62; 3.24) | 0.401 |
| TAG(54:4)-FA(20:3) | 1.49 (0.58; 3.84) | 0.404 |
| PC(18:0/18:1) | 1.34 (0.67; 2.67) | 0.404 |
| TAG(52:2)-FA(16:0) | 1.40 (0.63; 3.11) | 0.404 |
| TAG(54:4)-FA(20:4) | 1.38 (0.64; 2.99) | 0.410 |
| TAG(53:4)-FA(18:2) | 0.73 (0.35; 1.53) | 0.410 |
| PC(16:0/20:4) | 1.34 (0.66; 2.73) | 0.420 |
| FFA(20:1) | 1.33 (0.66; 2.69) | 0.422 |
| TAG(52:1)-FA(16:0) | 1.35 (0.63; 2.90) | 0.443 |
| TAG(52:4)-FA(18:2) | 1.36 (0.62; 3.02) | 0.446 |
| CE(20:4) | 1.34 (0.62; 2.91) | 0.454 |
| TAG(56:6)-FA18:1) | 1.36 (0.60; 3.09) | 0.457 |
| PC(14:0/18:2) | 0.72 (0.30; 1.73) | 0.461 |
| TAG(48:1)-FA(14:0) | 1.35 (0.60; 3.06) | 0.467 |
| TAG(49:1)-FA(15:0) | 1.34 (0.61; 2.95) | 0.468 |
| HCER(22:0) | 0.76 (0.36; 1.59) | 0.469 |
| TAG(52:3)-FA(18:1) | 1.37 (0.58; 3.24) | 0.469 |
| TAG(52:1)-FA(18:0) | 1.32 (0.62; 2.81) | 0.473 |
| PC(15:0/18:1) | 1.23 (0.70; 2.17) | 0.474 |
| PE(P-16:0/22:5) | 1.30 (0.64; 2.65) | 0.475 |
| PC(18:0/20:4) | 1.31 (0.62; 2.74) | 0.476 |
| DAG(16:0/18:2) | 0.73 (0.30; 1.75) | 0.480 |
| PC(16:0/22:6) | 1.38 (0.56; 3.42) | 0.483 |
| TAG(54:3)-FA(18:1) | 1.31 (0.61; 2.84) | 0.487 |
| TAG(50:2)-FA(16:0) | 1.37 (0.57; 3.29) | 0.488 |
| TAG(47:0)-FA(17:0) | 1.33 (0.59; 2.99) | 0.488 |
| TAG(44:0)-FA(14:0) | 1.30 (0.62; 2.73) | 0.491 |
| PC(18:0/22:5) | 1.30 (0.62; 2.71) | 0.492 |
| TAG(49:0)-FA(16:0) | 1.27 (0.64; 2.52) | 0.492 |
| PC(16:0/18:0) | 1.27 (0.63; 2.57) | 0.498 |
| TAG(54:5)-FA(18:2) | 1.27 (0.63; 2.55) | 0.502 |
| TAG(56:5)-FA(18:0) | 1.32 (0.59; 2.95) | 0.503 |
| FFA(18:3) | 1.26 (0.64; 2.45) | 0.503 |
| FFA(16:0) | 1.30 (0.59; 2.87) | 0.509 |
| PC(16:0/18:2) | 0.79 (0.40; 1.59) | 0.512 |
| PE(18:2/16:1) | 0.75 (0.32; 1.78) | 0.515 |
| PE(O-16:0/18:1) | 0.79 (0.39; 1.60) | 0.518 |
| PE(16:0/20:4) | 0.78 (0.38; 1.64) | 0.519 |
| TAG(54:2)-FA(18:0) | 1.28 (0.60; 2.75) | 0.521 |
| TAG(52:4)-FA(16:0) | 1.25 (0.63; 2.50) | 0.526 |
| CER(14:0) | 1.22 (0.66; 2.24) | 0.528 |
| CER(24:0) | 1.28 (0.59; 2.79) | 0.529 |
| PE(P-18:1/22:5) | 1.26 (0.61; 2.62) | 0.533 |
| PC(18:1/22:6) | 1.28 (0.59; 2.79) | 0.535 |
| SM(16:0) | 0.78 (0.35; 1.72) | 0.538 |
| FFA(20:3) | 1.32 (0.54; 3.26) | 0.543 |
| CER(20:0) | 1.28 (0.57; 2.86) | 0.545 |
| TAG(53:2)-FA(18:1) | 1.29 (0.57; 2.93) | 0.546 |
| TAG(52:5)-FA(18:2) | 1.32 (0.54; 3.22) | 0.548 |
| HCER(16:0) | 0.80 (0.39; 1.66) | 0.553 |
| SM(18:0) | 0.77 (0.32; 1.86) | 0.566 |
| PC(15:0/18:2) | 1.21 (0.62; 2.35) | 0.570 |
| DAG(18:1/18:1) | 1.22 (0.62; 2.39) | 0.573 |
| FFA(12:0) | 1.26 (0.56; 2.82) | 0.574 |
| PE(18:1/18:1) | 0.80 (0.36; 1.77) | 0.584 |
| CER(16:0) | 1.23 (0.59; 2.58) | 0.584 |
| TAG(54:4)-FA(16:0) | 1.22 (0.60; 2.50) | 0.584 |
| FFA(22:1) | 1.25 (0.56; 2.82) | 0.585 |
| TAG(56:7)-FA(20:3) | 0.82 (0.39; 1.70) | 0.586 |
| TAG(56:5)-FA(20:4) | 1.22 (0.58; 2.57) | 0.592 |
| TAG(46:1)-FA(16:0) | 1.24 (0.57; 2.67) | 0.592 |
| TAG(54:2)-FA(18:1) | 1.23 (0.57; 2.67) | 0.596 |
| PC(16:0/18:1) | 1.19 (0.62; 2.32) | 0.599 |
| TAG(50:3)-FA(16:0) | 1.24 (0.54; 2.83) | 0.610 |
| CER(22:0) | 1.24 (0.54; 2.82) | 0.610 |
| DAG(16:1/20:0) | 0.83 (0.41; 1.71) | 0.622 |
| TAG(52:3)-FA(16:0) | 1.22 (0.55; 2.73) | 0.624 |
| LPC(20:3) | 0.84 (0.41; 1.70) | 0.631 |
| TAG(56:5)-FA(18:1) | 0.83 (0.38; 1.80) | 0.632 |
| PE(P-18:0/22:5) | 1.20 (0.57; 2.51) | 0.633 |
| TAG(51:2)-FA(16:0) | 1.22 (0.51; 2.91) | 0.647 |
| PC(16:0/18:3 | 1.18 (0.57; 2.44) | 0.650 |
| TAG(51:2)-FA(18:1) | 0.82 (0.34; 1.97) | 0.659 |
| PE(P-16:0/18:2) | 0.85 (0.40; 1.79) | 0.662 |
| PC(18:0/18:2) | 0.85 (0.40; 1.79) | 0.669 |
| TAG(56:3)-FA(18:1) | 1.20 (0.50; 2.86) | 0.687 |
| PE(P-16:0/18:1) | 0.86 (0.42; 1.77) | 0.690 |
| PC(16:0/22:4) | 0.86 (0.40; 1.85) | 0.700 |
| PC(17:0/18:2) | 0.87 (0.41; 1.81) | 0.701 |
| TAG(52:2)-FA(18:2) | 0.88 (0.44; 1.73) | 0.702 |
| TAG(47:2)-FA(18:1) | 0.86 (0.40; 1.87) | 0.705 |
| PC(18:0/18:0) | 1.14 (0.56; 2.32) | 0.715 |
| LCER(24:0) | 0.88 (0.43; 1.80) | 0.728 |
| LPE(18:0) | 0.88 (0.43; 1.80) | 0.732 |
| PC(16:0/20:3) | 0.88 (0.41; 1.86) | 0.734 |
| PE(18:1/16:1) | 0.88 (0.42; 1.85) | 0.735 |
| LPC(18:0) | 1.15 (0.49; 2.68) | 0.748 |
| PC(16:0/16:0) | 1.11 (0.57; 2.19) | 0.752 |
| FFA(18:0) | 1.13 (0.53; 2.37) | 0.755 |
| FFA(20:4) | 0.89 (0.42; 1.89) | 0.757 |
| PE(P-18:0/22:6) | 0.88 (0.38; 2.03) | 0.763 |
| LPE(16:0) | 0.90 (0.44; 1.83) | 0.765 |
| PE(P-16:0/18:0) | 0.91 (0.45; 1.82) | 0.781 |
| LPC(18:1) | 0.89 (0.38; 2.09) | 0.784 |
| TAG(54:5)-FA(16:0) | 1.11 (0.51; 2.44) | 0.787 |
| SM(26:0) | 1.11 (0.50; 2.47) | 0.795 |
| CER(26:0) | 1.10 (0.52; 2.32) | 0.798 |
| PE(P-16:0/20:4) | 0.91 (0.43; 1.91) | 0.801 |
| CE(18:1) | 1.10 (0.52; 2.34) | 0.802 |
| PC(16:0/16:1) | 1.10 (0.53; 2.30) | 0.803 |
| PE(O-16:0/22:5) | 1.10 (0.51; 2.37) | 0.803 |
| SM(18:1) | 1.10 (0.51; 2.41) | 0.805 |
| PC(18:1/16:1) | 1.09 (0.54; 2.22) | 0.809 |
| TAG(50:0)-FA(18:0) | 1.08 (0.55; 2.13) | 0.820 |
| TAG(46:2)-FA(18:1) | 0.92 (0.44; 1.94) | 0.825 |
| LPC(16:0) | 1.09 (0.50; 2.38) | 0.828 |
| PC(18:1/18:1) | 1.08 (0.53; 2.20) | 0.832 |
| TAG(44:0)-FA(16:0) | 1.09 (0.49; 2.42) | 0.834 |
| TAG(55:5)-FA(20:4) | 1.08 (0.52; 2.23) | 0.836 |
| PC(16:0/20:2) | 0.94 (0.45; 1.94) | 0.858 |
| LPC(17:0) | 1.06 (0.54; 2.11) | 0.860 |
| PC(16:0/14:0) | 1.07 (0.52; 2.18) | 0.861 |
| PC(18:1/20:4) | 0.94 (0.46; 1.92) | 0.863 |
| PE(P-18:0/18:1) | 0.94 (0.45; 1.98) | 0.872 |
| FFA(18:4) | 0.95 (0.48; 1.91) | 0.890 |
| PC(18:2/20:3) | 0.96 (0.45; 2.06) | 0.915 |
| TAG(55:5)-FA(18:1) | 1.04 (0.49; 2.17) | 0.925 |
| FFA(22:6) | 0.97 (0.47; 2.03) | 0.940 |
| PC(17:0/18:1) | 0.97 (0.45; 2.11) | 0.945 |
| PC(18:0/20:3) | 1.03 (0.46; 2.30) | 0.947 |
| SM(14:0) | 0.98 (0.43; 2.23) | 0.954 |
| DAG(16:0/22:5) | 1.02 (0.47; 2.19) | 0.961 |
| TAG(52:0)-FA(18:0) | 1.02 (0.45; 2.31) | 0.963 |
| DAG(18:0/18:1) | 1.01 (0.51; 2.01) | 0.970 |
| PE(P-18:1/22:6) | 0.99 (0.44; 2.21) | 0.972 |
| TAG(54:1)-FA(18:1) | 0.99 (0.47; 2.06) | 0.972 |
| FFA(18:2) | 1.01 (0.46; 2.26) | 0.972 |
| TAG(56:7)-FA(20:4) | 1.01 (0.53; 1.92) | 0.977 |
| DCER(22:0) | 1.01 (0.47; 2.17) | 0.987 |
| TAG(47:0)-FA(14:0) | -* | -* |
| TAG(44:1)-FA(14:0) | -* | -* |
| TAG(54:2)-FA(16:0) | -* | -* |
| TAG(50:1)-FA(18:0) | -* | -* |
| TAG(51:2)-FA(17:0) | -* | -* |
| TAG(49:2)-FA(18:1) | -* | -* |
| TAG(49:1)-FA(17:0) | -* | -* |
| TAG(55:7)-FA(15:0) | -* | -* |
| TAG(46:2)-FA(14:0) | -* | -* |
| TAG(56:3)-FA(18:0) | -* | -* |
| TAG(56:3)-FA(20:1) | -* | -* |
| TAG(51:2)-FA(15:0) | -* | -* |
| Lipids were log transformed and mean scaled before logistic regression analyses. *Not estimable, all levels in non-responder group were below the detection limit. | | |

**Table S2**. Univariable association between oxylipin levels and prednisolone treatment response

| **Lipid** | **OR (95% CI)** | **P-value** |
| --- | --- | --- |
| 5-HEPE | 2.45 (1.07; 5.62) | 0.034 |
| 9-HOTrE | 0.31 (0.10; 0.96) | 0.041 |
| 10-HDHA | 2.13 (0.98; 4.64) | 0.058 |
| 20-HETE | 2.23 (0.86; 5.78) | 0.098 |
| 13- HODE | 0.46 (0.18; 1.18) | 0.107 |
| 14,15-DiHETE | 1.84 (0.80; 4.21) | 0.151 |
| EPA | 1.83 (0.78; 4.32) | 0.167 |
| 13-HOTrE | 0.40 (0.11; 1.47) | 0.168 |
| 17-HDHA | 1.76 (0.79; 3.92) | 0.170 |
| 4-HDHA | 1.71 (0.77; 3.77) | 0.185 |
| 12-HEPE | 1.65 (0.70; 3.87) | 0.251 |
| 14(S)-HDHA | 1.56 (0.71; 3.41) | 0.268 |
| AA | 1.52 (0.67; 3.46) | 0.318 |
| 5-HETE | 1.45 (0.69; 3.05) | 0.331 |
| 11-HETE11 | 1.46 (0.68; 3.16) | 0.333 |
| 19,20-DiHDPA | 0.77 (0.43; 1.39) | 0.381 |
| 18-HEPE | 1.35 (0.68; 2.66) | 0.388 |
| 12-HETE | 1.54 (0.57; 4.11) | 0.392 |
| 15-HEPE | 1.44 (0.62; 3.31) | 0.396 |
| AdA | 1.26 (0.57; 2.75) | 0.568 |
| n3-DPA | 1.26 (0.57; 2.75) | 0.568 |
| 8-HETE | 1.22 (0.59; 2.55) | 0.591 |
| DHA | 1.23 (0.57; 2.63) | 0.595 |
| TxB2 | 1.04 (0.50; 2.19) | 0.913 |
| LA | 1.02 (0.50; 2.08) | 0.957 |
| Lipids were log transformed and mean scaled before logistic regression analyses. | | |
